# Novel Treatment of 3D-Printed Short-Carbon-Fiber-Reinforced Polyamide (3D-SCFRPA66) Using Homogeneous Low-Potential Electron Beam Irradiation (HLEBI) and Ductility Enhancement

**DOI:** 10.3390/polym16233408

**Published:** 2024-12-03

**Authors:** Eiichi Miura, Helmut Takahiro Uchida, Taisuke Okazaki, Kohei Sagawa, Michael C. Faudree, Michelle Salvia, Hideki Kimura, Yoshitake Nishi

**Affiliations:** 1 KISTEC (Kanagawa Institute of Industrial Science & Technology), 705-1, Shimoimaizumi, Ebina 243-0435, Japan; e-miura@kistec.jp (E.M.); t-okazaki@kistec.jp (T.O.); west@tsc.u-tokai.ac.jp (Y.N.); 2 Graduate School of Engineering, Tokai University, 4-1, Kitakaname, Hiratsuka 259-1292, Japan; helmutuchida@tokai.ac.jp (H.T.U.); sagawa.kouhei@tsc.u-tokai.ac.jp (K.S.); kimura@tokai-u.jp (H.K.); 3 Doctoral Graduate School of Science & Technology, Tokai University, 4-1, Kitakaname, Hiratsuka 259-1292, Japan; 4 Faculty of Liberal Arts and Science, TCU (Tokyo City University), Yokohama 224-8551, Japan; 5 Laboratoire de Tribologie et Dynamique des Systemes (LTDS), ECL (Ecole Centrale de Lyon), CEDEX, 69134 Ecully, France; michelle.salvia@ec-lyon.fr

**Keywords:** 3D printing, polyamide 66, short carbon fiber, ductility, electron beam irradiation, tensile test

## Abstract

In short-carbon-fiber-reinforced polyamide 66 articles shaped by 3D printing (3D-SCFRPA66), the interfaces between printed layers are often susceptible to damage, and the composite is excessively brittle. Therefore, a novel treatment for 3D-printed short-carbon-fiber-reinforced polyamide (3D-SCFRPA66) using homogeneous low-potential electron beam irradiation (HLEBI) to enhance tensile properties was investigated. In 3D-SCFRPA66 samples, ductility was measured based on the following parameters: strain at tensile strength (corresponding to homogeneous deformation) (*ε*_ts_) and resistance energy to homogeneous deformation, a measure of toughness (*E*_hd_), which were both substantially increased. An HLEBI dose of 43.2 kGy at an acceleration potential of 210 kV for the finished 3D-SCFRPA66 samples increased the *ε*_ts_ and *E*_hd_ values from 0.031 and 1.20 MPa·m for the untreated samples to 0.270 and 6.05 MPa·m for the treated samples, increases of 771% and 504%, respectively. Higher HLEBI doses of 86, 129, or 215 kGy also increased the *ε*_ts_ and *E*_hd_ values to lesser degrees. Electron spin resonance (ESR) data in the literature show that HLEBI creates dangling bonds in Nylon 6. Since PA66 and Nylon 6 are constructed of C, N, and O and have similar molecular structures, HLEBI apparently severs the (-C-N-) bonds in the backbone of PA66, which have the lowest bond-dissociation energy (BDE) of ~326 to 335 kJ mol^−1^. This shortens the PA66 chains for higher ductility. In addition, for Nylon 6, X-ray photoelectron spectroscopy (XPS) data in the literature show that HLEBI reduces the N peak while increasing the C peak, indicating the occurrence of shortening chains via dangling bond formation accompanied by increases in crosslinking with carbon bonds. However, caution is advised, since HLEBI was found to decrease the tensile strength (*σ*_ts_) and initial elasticity ([d*σ*/d*ε*]_i_) of 3D-SCFRPA66. This tradeoff can possibly allow the HLEBI dose to be adjusted for the desired ductility and strength while minimizing energy consumption.

## 1. Introduction

A variety of factors, including bird strike, volcanic rock, hailstones, micrometeoroids, or space debris, can cause damage to aircrafts and space vehicles. Therefore, it is essential that their composite materials exhibit high strength and impact resistance to ensure maximum safety and reliability. Carbon-fiber-reinforced polymers (CFRPs) have been used in aerospace due to their lightweight properties, and one of the polymers used in CFRPA is thermoplastic (TP) polyamide (PA) [1,2].

PAs, or nylon materials [3], are semi-crystalline thermoplastics that contain nitrogen in their backbone [4]. The production of these materials comprises over 8 Mton per year, mostly from demand for Nylon 6 and Nylon 66 (PA6 and PA66), whose market shares are about 50% and 40%, respectively [4]. In nature, PAs occur as proteins, such as in silk or wool. To synthesize them artificially, processes such as step-growth polymerization or solid-phase synthesis are carried out to produce nylon fabric for articles such as swimwear, high-quality sportswear, and undergarments that require performance and comfort. They are also used for carpets, automotive components, and kitchen utensils. A large consumer is the transportation industry, which comprises about 35% of PA consumption [3]. Attractive properties of PA varieties include versatility, since it can be easily molded into different shapes and sizes; a good melting point from 160 to 350 °C, depending on the type; resistance to heat, chemicals, and wear; substantial elasticity; and durability, allowing it to be used for heavy-duty purposes.

However, to date, efficient recycling methods have not been developed for PA [4]. Instead, PA is reported to be burned for energy generation, which not only releases CO_2_ but also toxic nitrogenous NO_x_ greenhouse gases [4,5], which harm the human brain, and reduce the ozone layer [4]. N_2_O is a greenhouse gas that has a 265-times-higher global warming potential than CO_2_ [4]. Although beyond the scope of this study, the urgent need for developing methods of clean recycling of PA must be stressed here for a sustainable and healthy environment. Another disadvantage is that PA absorbs water easily, being hydroscopic. Hence, it needs to be dried prior to molding to avoid void formation. However, the 3D printing of the 3D-SCFRPA66 composite herein resulted in no observable voids.

**Figure 1:**
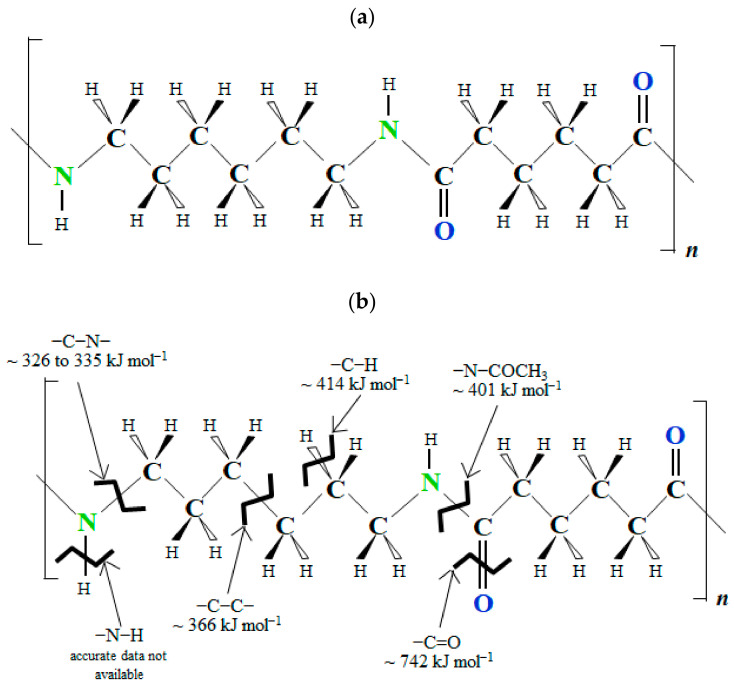
Rational formulae of (**a**) thermoplastic polyamide 66 and (**b**) indicating bond-dissociation energies (BDEs) [6,7,8,9]. Carbon, nitrogen, oxygen, and hydrogen symbols are depicted in black, green, blue, and black (smaller letters). For the (-N-H) bond, accurate data have not been found [9].

Figure 1a illustrates the rational formula for PA66, showing the monomer unit composed of two sub-chains, each with six carbons, as well as two nitrogen atoms and two oxygen atoms.

Lately, 3D printing, also known as additive manufacturing, has been a widely researched area [1,10,11,12,13]. Previous research has indicated that if durable 3D polymer composites can be printed successfully, they can contribute to increased recyclability for a more sustainable environment and circular economy [11]. Three-dimensional printing is the construction of a 3D object from a computer-aided design (CAD) or another digital 3D model. This process involves the deposition, joining, or solidification of material under computer control, typically in a layer-by-layer manner. Advantages of 3D printing include high-mix, low-volume production; the ability to make complex shapes that cannot be formed without cutting or grinding; and the minimization of waste material. For composites, filaments can contain long or short fibers [12]. However, there are disadvantages to 3D printing. First, parts are not pressurized for consolidation, as in injection or compression molding, and therefore there could be weak bonding between printed layers [1,13]. For TPs, Fused Deposition Modeling (FDM) is not suitable for final products due to the low strength of TP resin-printed surfaces. Weak fracture toughness represents a significant challenge [13] due to the presence of surface cracks between solidified and liquid layers, which are caused by the entanglement of polymers and molecular forces [1]. It follows that the interface between 3D-printed layers is often susceptible to damage [1,13], and the strong bonding of CF and the polymer is important [14]. Hence, researchers have investigated interlaminar fracture toughness, *G*_XC_, for 3D-CFRPA [13]. Measurements of long-fiber 3D-CFRPA samples have been carried out under Mode I, II, and Mixed Mode I–II by double cantilever beam (DCB), end-loaded split (ELS), end-notched flexure (ENF), and mixed-mode bending (MMB) tests. Katalagarianakis et al. (2023) [13] found at crack initiation that the interlaminar fracture toughness for Mode I, *G*_1C_, was 1.5 kJm^−2^. For Mode II, the *G*_2C_ values were found to be 2.1 kJm^−2^ from the ELS test, and 1.8 kJm^−2^ from the ENF test, while for Mixed Mode I–II, *G*_(1-2)C_ was 1.0 kJm^−2^ at G_II_/G_total_ = 0.5 [13].

Lately, research on the 3D printing of non-reinforced PA resin [15,16,17,18,19] and 3D-printed CFRPA composites [12,13,20,21,22,23,24,25,26,27,28,29,30,31,32,33,34,35] has been gaining much attention, mostly in the last two years. Papers for non-reinforced 3D-PA resin include studies on the following: 3D printing of PA for reverse-osmosis membranes used for water desalination [15]; comparison between selective laser sintering and multijet fusion 3D-printing processes of PA12 [16]; performance optimization of multijet fusion processes for PA12 [17]; evaluation of mechanical properties of 3D-printed PA6 polymers [18]; and comparison of non-reinforced PA6 resin with CFRPA and glass-bubble-reinforced PA12 [19]. Those for 3D-printed CFRPA composites include [19] along with studies on the following: CF hybrid composites [12]; 3D-printed CFRTPA gears and their fatigue optimization [20]; moisture absorption studies [21,23]; tailorable rigidity and energy absorption [22]; hydrophobic coatings [23]; flexible broadband microwave absorber [24]; crushing behavior [25]; 3D-printed CFRPA thin films [26]; hygrothermal aging [27]; impact behavior [28]; interlaminar fracture toughness, G_1C_, G_2C_, and mixed-mode G_1,2C_ [13]; 3D-printed CFRTA with recycled CF [29]; the effect of wt.% CF, temperature, printing speed, and printed-layer thickness on the tensile stress of 3D-CFRPA [1]; and the fabrication-induced interface effect on tensile properties [35]. It has been reported that the interlaminar interface between 3D-printed layers is often susceptible to damage [13].

However, for 3D-CFRPA, as far as the authors know there has been no research on applying a treatment that penetrates into the surface of finished samples to increase cohesion and interlaminar toughness between printed layers and at the CF/PA interface. Homogeneous low-voltage electron beam irradiation (HLEBI) applies a low-energy curtain beam of electrons that penetrates into the surface and generates dangling bonds [36,37]. The dangling bonds create nano-compressive stress sites in polymers, by the repulsive force of the lone-pair electrons generated to increase strength in the polymers themselves, and to increase the adhesion force of the difficult-to-adhere thermoplastic (TP) with CFs, both of which have inert surfaces [37]. For 3D-CFRPA, if HLEBI can penetrate through the outer surface printed layer and into the second layer to activate the interface between them, it is possible to improve the mechanical properties, such as the ductility and interlaminar fracture toughness of the composite as a whole. Therefore, in order to fabricate parts that are less brittle with higher toughness, along with enhancing adhesion between laminated polymer layers and the CF/TP interface, we employ the novel method for 3D-SCFRPA66 of HLEBI treatment. HLEBI has a history of improving various properties of short-glass-fiber GFRP, long-fiber CFRPs, and glasses such as silica glass [37,38,39,40]. HLEBI often enhances the deformation resistivity (elasticity) of a polymer [41]. Furthermore, for a short-carbon-fiber-reinforced polyetheretherketone (SCFRPEEK) matrix composite, it is also possible to enhance the CF/TP interfacial strength by surface activation, which is generated by charging and dangling bond formation, as well as atom migration at the interface [42]. As for the CF component, HLEBI at an optimum dose has been found to increase the tensile stress of CF by about 20% [39].

Figure 1b indicates the bond-dissociation energies (BDEs) for PA66 whose values for (-C-N-), (-C-C-), (-C-H), (-C-O-), (-N-COCH_3_), and (-C=O) are reported to be ~326–335, ~366, ~414–378, ~401, and ~742kJmol^−1^, respectively, [6,7] the weakest being (-C-N-) at ~326–335 kJ mol^−1^. Note that, to date, for the (-N-H) bond, accurate data for BDE have not been experimentally determined [8,9]. This is because molecules with the (-N-H) bond are reported to be unstable and hard to obtain in the gas phase; the (-N-H) bond has a high sensitivity to its environment, and its vibration is reported to be anharmonic [9]. HLEBI acts to cut chemical bonds in materials generating dangling bonds in the form of lone-pair electrons with the highest severing probability at low BDE sites [38,42]. It follows the (-C-N-) bond has the lowest BDE at ~326–335 kJmol^−1^, and resides on the polyamide 66 chain backbone: thus, HLEBI will act to shorten the PA polymer chains to increase ductility. Hence, we apply the novel use of HLEBI treatment to 3D-SCFRPA66, with the goal of increasing the ductility and toughness of the finished part.

Caution must be advised, however, that enhancements in mechanical and physical properties were only achieved when the optimum HLEBI dose was administered. In general, too low a dose will not reach the optimum, but too high a dose will proliferate excess dangling bonds, severing chains too short, weakening the material. Nevertheless, we present results on lowering the undesired brittleness and weak adhesion [1,13] of the finished 3D-printed SCFRPA66 specimens by HLEBI with the aim of obtaining wider use for practical applications.

## 2. Experimental Procedure

### 2.1. Preparation of 3D-SCFRPAA66

**Figure 2:**
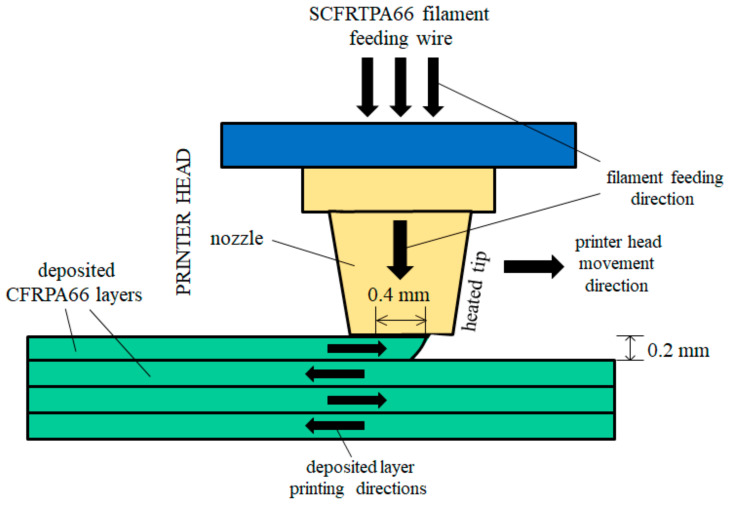
Schematic of 3D-printing process (not to scale).

**Figure 3:**
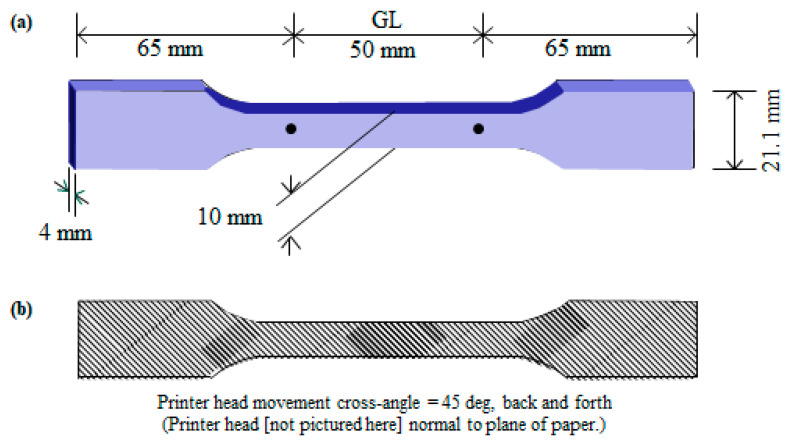
Schematic of (**a**) dog-bone specimen dimensions; and (**b**) printer cross angle head movement of 45 deg angle with respect to specimen length.

Composite samples (short-CF-reinforced polyamide 66 (SCFRPA66: Ultrafuse^®^ PAHT CF15, BASE Ltd., Tokyo, Japan) were shaped by a 3D-printer (Kobra Neo, Anycubic Ltd. Tokyo: see Figure 2). The feeding wire was a composite prepared by short straight CFs (6 μm diameter; 85 +/− 15 μm length) wrapped by PA66, made molten, then cooled. The SCFRPA66 filament was heated and melted at the tip and cooled on the deposited samples. The printing temperature at the tip was 260 °C, which is about the same as the melting point of PA66 [43]. The glass transition temperature, *T*_g_, of PA is reported to be 70 to 90 °C [44]. The nozzle was perpendicular to the specimen surface. The head moved along the cross-length of the specimen programmed to the dog-bone shape, building it up layer by layer with cross angle of 45° (see Figure 3). There was no mold used to form the dog-bone shape. The flow rate of the polymer out of the nozzle was 50 mm/s. The nozzle hole diameter was 0.4 mm, while the distance between the nozzle and the printing surface was 0.2 mm. The depositing speed was precisely indicated by both the depositing periods of one layer (3 min) and of a dog-bone sample (56 min). Figure 3 illustrates the 3D-SCFRPA66 dog-bone specimen dimensions: a gauge length, thickness, width, and total length of 50 mm, 4 mm, 10 mm, and 180 mm, respectively. Since the sample thickness is 4 mm, each deposited layer is calculated to be ~214 mm. The CF volume fraction (V_f_) was approximately 15%. There was no infill. SEM observation showed no air gaps in the composite filament before printing; and none in dog bone samples after printing (before tensile testing). In this experiment, gels were not used and were not observed.

### 2.2. Tensile Tests

The mechanical properties of the 3D-SCFRPA66 samples were evaluated by an Instron-type tensile tester (Instron 5800R 5582, Instron Japan Co. Ltd., Kawasaki, Japan) with the JIS K 7161 standard [45]. The strain rate (load speed) was 1 mm/min. The HLEBI dose levels were 43, 86, 129, and 215 kGy (see next section), along with the untreated condition. Note that only one sample was tested for each HLEBI dose level due to constraints in available materials and equipment time. Since the experimental results showed the HLEBI-treated samples had a substantially higher fracture strain than that of the untreated (the 43 kGy sample had > 700% increase), it is deemed the HLEBI had a significant effect on increasing ductility. All samples were fabricated the same day in a constant temperature and humidity environment. Although fracture occurred close to the beginning of the taper, it occurred in the same position in each sample in the thin section of the dog-bone samples. Reproducible data have been obtained in the literature that HLEBI significantly increases ductility in 3D-SCFRPA samples [46].

The tensile parameters evaluated were (1) strain (elongation) at maximum tensile strength corresponding to homogeneous deformation (*ε*_ts_); (2) fracture strain (*ε*_f_); (3) the difference corresponding to partial plastic deformation (Δ*ε*_ppd_ = *ε*_f_ − *ε*_ts_); and (4) the resistance energy to homogeneous plastic deformation (*E*_hd_) which corresponds to area under the stress–strain curve and is a measure of toughness. Fracture stress (*s*_ts_) and Young’s modulus (d*s*/d*e*)_i_ were also evaluated. Strain values are based on the 50 mm gauge length.

### 2.3. Homogeneous Low Voltage Electron Beam Irradiation (HLEBI)

Samples were homogeneously irradiated using an electron-curtain processor (Type LB250/15/180L), Energy Science, Inc., Woburn, MA, Iwasaki Electric Group, Ltd., Tokyo, Japan) [37] with the electron beam through a titanium thin film window attached to a vacuum chamber, ~240 mm in diameter. A tungsten filament in the vacuum was used to generate the electron beam at an acceleration potential of 210 kV and an irradiating current of 2.68 mA. To prevent oxidation, the samples were kept in a 1 atom N_2_ atmosphere with a residual O2 concentration below 300 ppm. The N_2_ gas molar flow rate was 89 Nmin^−1^. The distance between the sample and the Ti window was 30 mm. Samples were transported in a 200 × 150 mm aluminum plate holder on a conveyor at a speed of 10 mmin^−1^. One sweep going one way was 42.3 kGy for the short time of 0.20 s to avoid excess heating of the sample. Repetitive applications to both side surfaces were applied to achieve the desired dose of HLEBI, with a gap interval of 20 s between each sweep. The resulting EB dosage (dose = 0.216(I/S)N) was proportional to the yield value determined from the irradiation current, I (mA) conveyor speed, S (mmin^−1^), and number of irradiations, *N*. The yield value was calibrated by FWT nylon dosimeters (Far West Technology, Inc. 330-D South Goleta, CA 93117, USA).

To determine the HLEBI penetration depth, *D*_th_, into the sample surface, the following equation with a mean density, *ρ* (kgm^−3^), and irradiation potential at the specimen surface, *V*_c_, at 210 kV is used [47]:

*D*_th_ = 66.7*V*^5/3^/*ρ* (1)

The density of CF and PA are reported to be 1760 [48] and 1140 kgm^−3^ [6]; therefore, their penetration depths are calculated to be 208 μm (CF) and 322 μm (PA66), respectively. Each deposited 3D-SCFRPA layer is approximately 214 μm in thickness. Since the CF volume fraction (*V*_f_) was approximately 15%, the *V*_c_ setting of 210 kV is considered to be sufficient to penetrate through the specimen outer layers and into the second layer to activate the interface between the outer and second layers.

## 3. Results

### 3.1. Influence of Short CF Addition and 3D Printing: Comparing 3D-SCFRPA66 with PA66 Resin

**Figure 4:**
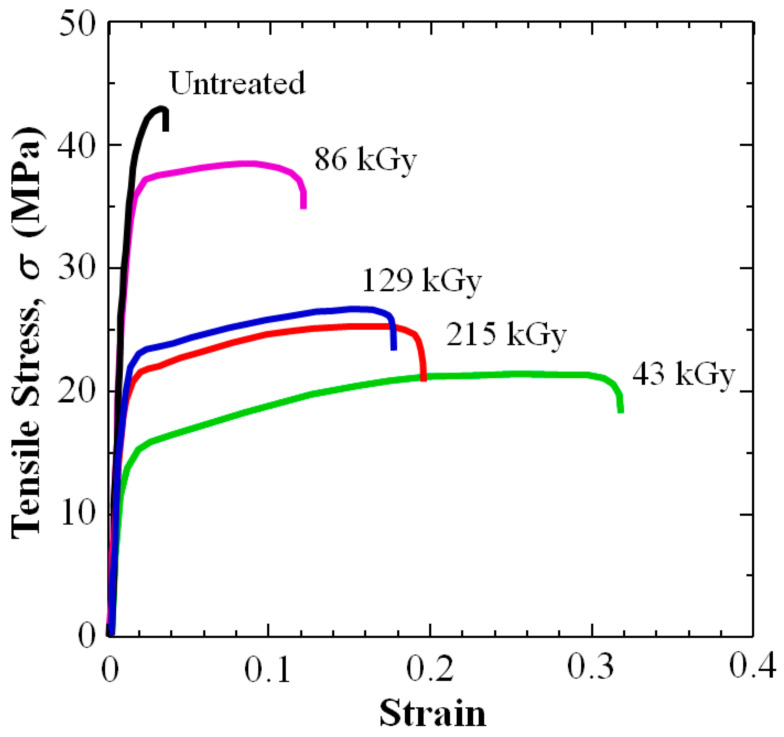
Tensile stress–strain curves of 3D-SCFRAP66 with each dose of 210 kV-HLEBI.

Figure 4 shows the 3D-SCFRPA66 untreated sample (black line) exhibited a relatively brittle fracture with tensile strength (*σ*_ts_), strain (*ε*_ts_), fracture strain (*ε*_f_), and Young’s modulus [d*σ*/d*ε*]_i_ at 43 MPa, 0.031, 0.034, and 4.24 GPa, respectively. Note that *σ*_ts_, *ε*_ts_, and *ε*_f_, were 31 to 95%, 61%, and 1665% lower than that reported for PA66 resin itself at 62 to 83 MPa [6], ~0.05 (modified PA66) [49], and 0.60 [50]. The [d*σ*/d*ε*]_i_ of 3D-SCRRPA66 at 4.24 GPa is 34% higher than that reported for PA66 at 2.8 [50].

### 3.2. Effect of HLEBI on Ductility of 3D-SCFRPA66

Figure 4 shows applying HLEBI at doses of either 43, 86, 129, or 215 kGy to both sides of the 3D-SCFRPA66 samples significantly increases ductility as evidenced by an increase in ε_ts_, ε_f_, and Δε_ppd_ over those of untreated samples. Notably, the toughness parameter, E_hd_ (MPa·m), is also substantially increased. However, as expected, the HLEBI decreases σ_ts_ (MPa) and [dσ/dε]_i_ (GPa).

Table 1 and Table 2 list values and percentage changes, respectively. Particularly, the 43 kGy HLEBI dose appears to be the optimum out of the doses tested, increasing ε_ts_, ε_f_, Δε_ppd_, and E_hd_ by 771%, 829%, 1433%, and 404%, respectively, from 0.031, 0.034, 0.003, and 1.20 MPa·m (untreated) to 0.270, 0.316, 0.046, and 6.05 MPa·m (43 kGy). Since the increase is substantial, this result is considered valid. The 215 kGy dose increases ε_ts_, ε_f_, Δε_ppd_, and E_hd_ by 416%, 471%, 1033%, and 289%, respectively, over that of untreated samples to 0.160, 0.194, 0.034, and 4.67 MPa·m (43 kGy).

**Figure 5:**
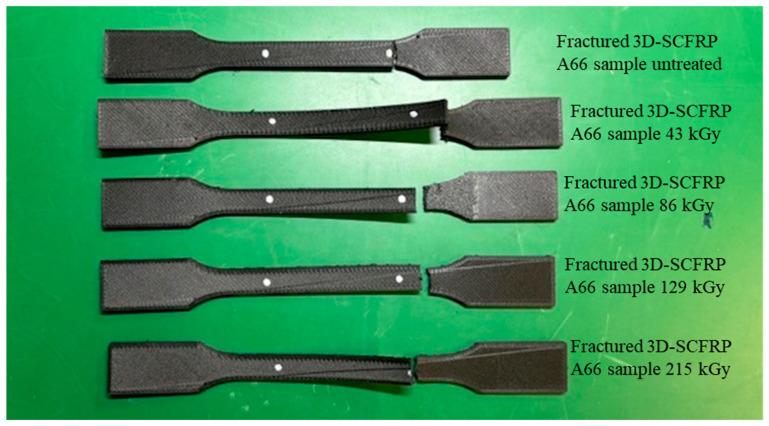
Photograph of fractured 3D-SCFRPA66 samples untreated and treated by 210 kV-HLEBI.

Figure 5 shows a photograph of the fractured 3D-SCFRPA66 dog-bone samples. Fracture is observed to occur in the same position in each sample, outside of the 50 mm gauge length, but in the thin section. As mentioned earlier, there was one sample available for each HLEBI dose. Because the HLEBI-treated samples had a substantially higher ε_ts_ than that of the untreated sample (the 43 kGy sample had > 700% increase), the result that HLEBI increases toughness and ductility in the 3D-SCFRPA66 samples can be considered valid.

Thus, the HLEBI apparently improves the ductility in the homogeneous elastic and heterogeneous plastic regions.

## 4. Discussion

### 4.1. Effect of HLEBI Increasing Tensile Parameters,ε_ts_, ε_f_, and Δε_ppd_ and Toughness, E_pd_ of 3D-SCFRPA66

**Figure 6:**
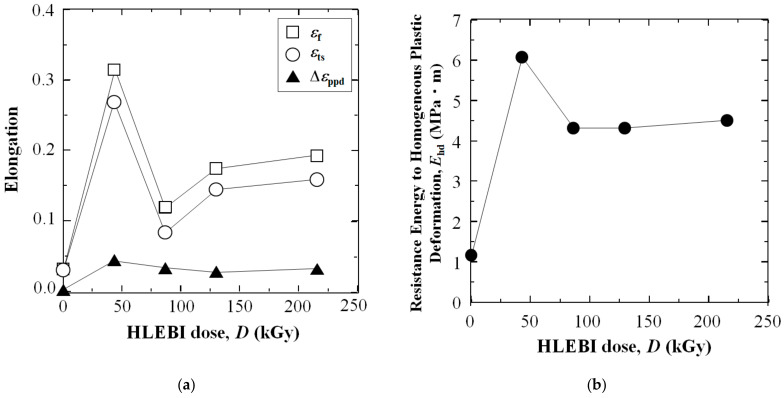
Plots of (**a**) change in tensile parameters: elongation ε_ts_, ε_f_, and (Δε_ppd_ = ε_f_ − ε_ts_); along with (**b**) the toughness E_hd_ (MPa·m) of 3D-SCFRPA66 against HLEBI dose.

**Figure 7:**
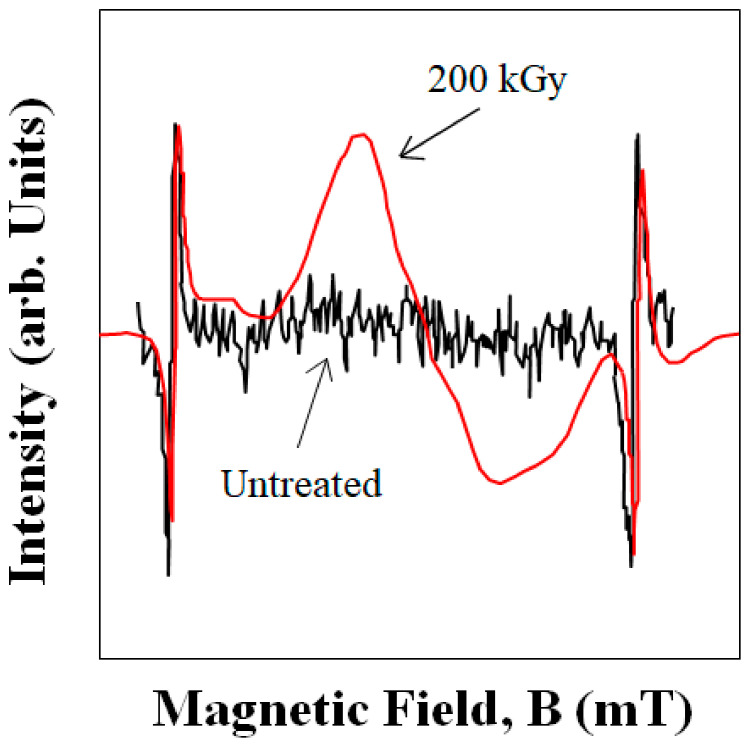
ESR signals for 200 kGy HLEBI-treated and untreated Nylon 6 samples. Adapted from Nishi et al. (2008) [51].

Figure 6a,b show (a) the change in the tensile parameters: elongation ε_ts_, ε_f_, and Δε_ppd_; and (b) the toughness, E_pd_, of 3D-SCFRPA66 against the HLEBI dose. Namely, applying a 43 kGy dose results in substantial strain enhancements of ε_ts_ and ε_f_, which are in turn decreased sharply at 86 kGy, and then are increased gradually again from 129 to 215 kGy. Electron spin resonance (ESR) data in the literature show HLEBI creates dangling bonds in Nylon 6 [51] as evidenced by the sharp peak generation shown in Figure 7. Here, dangling bonds were not observed by ESR in untreated Nylon 6, but were generated by HLEBI at 200 kGy. ESR detects dangling bonds by detecting lone-pair electrons which emit a magnetic field. Each lone pair has a spin of +1/2 or −1/2 [38]. Thus, HLEBI cuts the bonds in the Nylon 6. Since PA66 and Nylon 6 are constructed of C, N, and O and have a similar molecular structure, HLEBI apparently severs the (-C-N-) bonds in the backbone of PA66 (see Figure 1), which are at the lowest bond-dissociation energy (BDE) in the PA66 chain at ~ 326–335 kJmol^−1^ [6,7]. This shortens the chains and increases ductility. Thus, by applying HLEBI at a 43 kGy dose, a state of matter can be achieved for the highest strain and toughness properties.

The properties of the composite surface and bulk can differ. The HLEBI penetration depth, *D*_th_, is calculated to be 322 μm for the PA66, and 208 μm for CF. With the 14% *V*_f_ of CF, the *V*_f_ of the PA66 is 85%; thus, it is assumed the electron beam can easily penetrate to a 322 μm depth and activate the interface between the outside and second-to-outside layers, while activating the highly conductive CFs. A skin/core/skin sandwich structure is generated across the 4 mm specimen thickness of 0.322/3.36/0.322 mm having ductile/brittle/ductile properties. With the dangling bond generation, the ductility enhancement of the skin will be accompanied by increased CF/TP interfacial adhesion. The highly conductive CF will apparently transfer charge deeper into the bulk to a certain degree, making the brittle/ductile gradient more gradual. It appears the ductile “skins” dominate the deformation mechanism. The investigation of specimen thickness effects was beyond the scope of this study.

**Figure 8:**
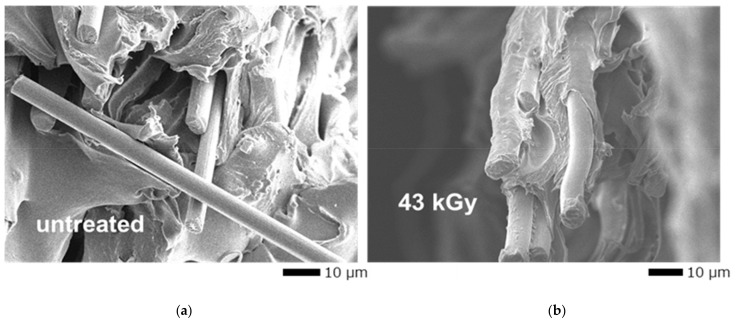
SEM micrographs of 3D-SCFRPA66: (**a**) untreated; and (**b**) treated with 43 kGy-HLEBI.

The ductility (ε_ts_, ε_f_, and Δε_ppd_) and toughness (E_pd_) improvements can be explained by four factors. First, the severing of PA66 polymer chains would allow more freedom of movement between the shortened polymeric chains to withstand higher strains. Secondly, lone-pair electrons generated in the matrix may have bonded with CFs more efficiently. This has been found for Ti-CF-TP joints connected by a CF insert: when bare CF was activated with HLEBI prior to dipping in molten TP, the tensile strength of the joint was increased by increased adhesion between the difficult-to-adhere CF and TP [52]. Thirdly, by HLEBI, dangling bond generation creates repulsive forces between outer shell electrons within the polymer matrix [38], increasing the compressive stress on the CF and raising the adhesion force. Fourthly, since HLEBI relaxes the polymeric chains, it is also difficult for brittle cracks to generate and propagate at the CF/TP interface. This is supported by the SEM photomicrography in Figure 8 showing increased CF/PA66 matrix adhesion in the fractured 43 kGy sample compared with that of the untreated sample. To estimate the quantity of increase in PA66/CF interfacial adhesion sites by HLEBI-treated 3D-SGFRPA over untreated samples, plots of dangling bond density (×10^−12^ spins/mm^3^) vs. penetration depth, *D*_th_, into the specimen must be determined. This has previously been performed with polyetheretherketone (PEEK) by meticulously cutting successive layers and analyzing them by ESR in [42].

In addition, Table 3 shows previously reported X-ray photoelectron spectroscopy (XPS) results of HLEBI-activated Nylon 6 [51]. HLEBI was found to decrease the intensity of N peaks in Nylon 6. This would be due to dangling bond formation, since the XPS peak height indicates the detection of bonds. This result is congruent with that reported for ESR analysis, and that predicted by BDEs (see Figure 1b) that the atomic bonding of (-C-N-) has the weakest bonding link in the PA66 backbone, and thus would have the highest probability of breaking.

On the other hand, HLEBI was reported to increase the C peak of XPS in Nylon 6, indicating the formation of (-C-C-) bonds [51]. This would indicate the presence of crosslinking and agrees with other studies of HLEBI on polymers [53].

### 4.2. Influence of HLEBI on Tensile Strength and Young’s Modulus of 3D-SCFRPA66

**Figure 9:**
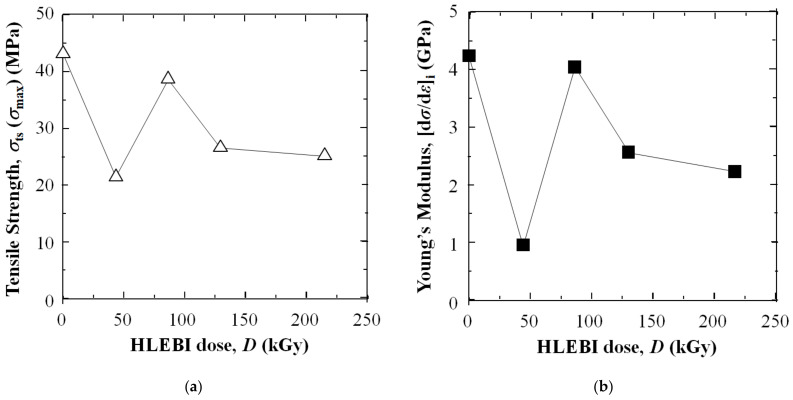
Changes in (**a**) tensile strength and (**b**) initial elasticity (Young’s Modulus), [dσ/dε]_i:_ (GPa) in 3D-SCFRPA66 against HLEBI dose.

However, Figure 9a,b show there is a tradeoff that increasing ductility and toughness decrease tensile strength and Young’s modulus in the 3D-SCFRPA66 for all doses tested: 43, 86, 129, and 215 kGy. The 43 kGy-HLEBI dose largely decreases the σ_ts_ to 21.5 MPa, 50% lower than that of the untreated samples; while decreasing [d*σ*/d*ε*]_i_ 77% from 4.94 to 0.96 GPa as shown in Figure 4 and Table 1 and Table 2.

### 4.3. Effects of Higher HLEBI Doses of 86, 129, and 215 kGy

But interestingly, Figure 9a,b show the 86 kGy dose resulted in a recovery of σ_ts_ and (ds/de)_i_ over that of the 43 kGy dose. The 86 kGy dose showed an increased σ_ts_ and a [d*σ*/d*ε*]_i_ of 39.0 MPa and 4.04 GPa over those of the 43 kGy dose at 21.5 MPa and 0.96 GPa, but decreased ductility, ε_ts_ and ε_f_, and toughness, E_hd_. This can be explained by crosslinking in the “skins” by the higher 86 kGy dose acting to increase the tensile strength and brittleness.

However, at 129 kGy, ductility again is increased with a decrease in σ_ts_ and [d*σ*/d*ε*]_i_ compared with that of 86 kGy as shown in Figure 6a and Figure 9a,b. This was apparently by the higher 129 kGy HLEBI increasing the dangling bond generation that acts to sever bonds in the crosslinked structure, but creating stronger CF/PA66 adhesion. An increase in the HLEBI to 215 kGy further increases the ductility over that of 129 kGy by increasing the dangling bonds. These phenomena indicate that by using HLEBI, it is possible to tailor the tensile properties of 3D-SCFRPA.

### 4.4. Future Scope

The future scope in detail is proprietary but should include fabricating and testing a larger number of 3D-SCFRPA specimens and examining the effects of intermediate HLEBI dose levels, head speed, layer thickness [30], and testing temperature [54]. If possible, for the future scope it is recommended to treat the CFs directly with HLEBI prior to producing the 3D-SCFRTA filament feeding wire. In an interlayered composite of 3 CF plies alternating between 4 TP PC plies, designated [PC]_4_[CF]_3_, applying a 215 kGy HLEBI dose directly to the CF plies prior to lamination assembly and hot-pressing increased the three-point bending strength 25% at the median fracture probability, *P*_f_ = 0.50 [37].

## 5. Conclusions

In short-carbon-fiber-reinforced polyamide 66 articles shaped by 3D printing (3D-SCFRPA66) the interfaces between 3D-printed layers are often susceptible to damage and the composite is excessively brittle. In order to improve on this, homogeneous low potential electron beam irradiation (HLEBI) to both sides of 3D-SCFRPA66 samples was found to significantly increase the ductility according to the following parameters: strain at tensile strength (corresponding to homogeneous deformation) (*ε*_ts_); as well as the resistance energy to homogeneous deformation, a measure of toughness, *E*_hd_.

1. Applying HLEBI at doses of either 43, 86, 129, or 215 kGy to both sides of the 3D-SCFRPA66 samples significantly increases ductility (ε_ts_, ε_f_, and Δε_ppd_) over untreated samples. Notably, the toughness parameter E_hd_ (MPa·m) is also substantially increased. Particularly, the 43 kGy HLEBI dose appears to be the optimum out of the doses tested, increasing ε_ts_, ε_f_, Δε_ppd_, and E_hd_ by 771%, 829%, 1433%, and 404%, respectively, from 0.031, 0.034, 0.003, and 1.20 MPa·m (untreated) to 0.270, 0.316, 0.046, and 6.05 MPa·m (43 kGy).
2. Applying a 43 kGy dose results in the substantial strain enhancements of ε_ts_, ε_f_, and Δε_ppd_, which are in turn decreased sharply at 86 kGy, and then are increased gradually again from 129 to 215 kGy. Electron spin resonance (ESR) data in the literature show HLEBI creates dangling bonds in Nylon 6 as evidenced by sharp peak generation. Thus, since PA66 and Nylon 6 are constructed of C, N, and O and have a similar molecular structure, HLEBI apparently severs the (-C-N-) bonds in the backbone of PA66 (see Figure 1), which are at the lowest bond-dissociation energy (BDE) in the PA66 chain at ~326–335 kJmol^−1^. This shortens the chains and increases ductility. Thus, by applying HLEBI at a 43 kGy dose, a state of matter can be achieved for the highest strain and toughness properties.
3. However, caution is advised since there is a tradeoff that increasing ductility and toughness decrease the tensile strength and Young’s modulus in the 3D-SCFRPA66 for all doses tested: 43, 86, 129, and 215 kGy. The 43 kGy-HLEBI dose largely decreases the σ_ts_ to 21.5 MPa, 50% lower than that of the untreated samples, while decreasing [d*σ*/d*ε*]_i_ 77% from 4.94 to 0.96 GPa. This tradeoff can possibly allow the HLEBI dose to be adjusted for desired ductility and strength, while minimizing energy consumption.

## Figures and Tables

**Table 1:** Effect of HLEBI treatment on 3D-SCFRAP66 tensile parameters compared with untreated (shading and italics indicate decreasing values).

Treatment	*ε*_ts_	*ε*_f_	Δ*ε*_ppd_	*E*_hd_ (MPa·m)	*σ*_ts_ (MPa)	[d*σ*/d*ε*]_i_ (GPa)
Untreated	0.031	0.034	0.003	1.20	43.0	4.24
43 kGy	0.270	0.316	0.046	6.05	*21.5*	*0.96*
86 kGy	0.085	0.120	0.035	4.30	*39.0*	*4.04*
129 kGy	0.147	0.176	0.029	4.30	*26.0*	*2.56*
215 kGy	0.160	0.194	0.034	4.67	*25.0*	*2.23*

**Table 2:** Percentage change in 3D-SCFRAP66 tensile parameters from HLEBI treatment compared with untreated samples from the data in Table 1.

Treatment	*ε*_ts_	*ε*_f_	Δ*ε*_ppd_	*E*_hd_ (MPa·m)	*σ*_ts_ (MPa)	[d*σ*/d*ε*]_i_ (GPa)
Untreated	-	-	-	-	-	-
43 kGy	771%	829%	1433%	404%	*−50%*	*−77%*
86 kGy	174%	253%	1067%	258%	*−9%*	*−5%*
129 kGy	374%	418%	867%	258%	*−40%*	*−40%*
215 kGy	416%	471%	1033%	289%	*−42%*	*−47%*

**Table 3:** Summary of XPS data of HLEBI treatment of Nylon 6 from Nishi et al. (2008) [51].

XPS Peak Intensity	O	N	C
Unt’d ==> HLEBI	decreased and increased	decreased	increased

## Data Availability

Data are available from the corresponding author upon request.
